# Observations and Experiments on a New Mode of Treating Fractures of the Leg and Fore Arm; Especially Compound Fractures

**Published:** 1831-10

**Authors:** 


### BIBLIOGRAPHICAL NOTICE.

*Observations and Experiments on a new Mode of Treating Fractures of the Leg and Fore Arm; especially Compound Fractures.* By Wm. Beaumont, Member of the Royal College of Surgeons, and Surgeon to the Farringdon Dispensary.—8vo. pp. 31. London.

Although this "new mode" of treating fractures may never have been employed in this country, it is in reality a very old one, as our readers will find by referring to our Journal for February 1829, p. 175, where an account is given of the "Arabian Mode of curing Fractured Limbs." The Orientals will never consent to the amputation of a limb: their practice is to lay the fractured limb on an oiled mat, after reducing the bones, and then enclose it in a case of gypsum, or plaster of Paris. Baron Larrey has lately tried a somewhat similar plan, with a certain degree of success, but its advantages have not been sufficiently striking to lead to its general adoption among the French surgeons. The Baron does not make use of a case of plaster of Paris, but surrounds the limb with bandages which have been soaked in some glutinous matter; and thus nearly as firm an envelope is formed for it as would be constituted by the plaster of Paris.

The plan proposed by Mr. Beaumont, and which he conceives to originate with himself, is precisely the same in principle as that we have formerly mentioned as peculiar to the Arabian surgeons. It appears, however, that the latter applied their case of plaster in such a manner as to render it more eligible and safer in a greater number of cases of fracture, and especially of compound fracture, than the mode recommended by Mr. Beaumont; for they so contrived its application, that blood or matter found a ready exit; and they could also examine the state of the limb during the progress of the cure, without in the least altering the position of the fractured bones. These advantages are not to be derived from Mr. Beaumont's plan, and consequently it must be more limited in its application: he does not, indeed, recommend it when the contusion or laceration of the soft parts is very extensive. The mode Mr. B. proposes of treating fractures of the leg and fore arm, especially compound fractures, consists, for the most part, "in incrusting the limb in plaster of Paris." He considers it very superior to the use of splints, for it necessarily prevents any motion of the fractured bones; which important advantage is certainly not to be obtained, in many cases, by the ordinary means of securing the injured limb.

"Splints can only prevent retraction by their lateral pressure, which, to effect that purpose, must be great in proportion to the obliquity of the fracture, and the force with which the muscles may be disposed to contract: but by the mode of treatment I propose, no lateral pressure is necessary to prevent retraction; for the two extremities of the fractured limb are gently grasped, as it were, and held at an unvarying distance from each other: wherefore, if the limb be but extended to, and maintained of its proper length, during the deposition and hardening of the plaster, it is utterly impossible that, on the recovery of the patient, his limb should be found in the least shortened. And, in like manner, with regard to the preservation of its form: let the distal portion of the limb be held during the consolidation of the plaster, in a position neither everted nor inverted, nor bent upon the proximal portion; and of necessity it must continue so, as long as the incrustation remains unbroken. In this manner, the shortening of a fractured leg or fore arm, its eversion or inversion, may be prevented, whilst the pressure employed need be neither painful nor injurious: in fact, it can be no more than that made by the bandage put around the limb previously to the incrusting of it in plaster of Paris. It is true that, where the soft parts have been much bruised, inflammation may supervene, disposing the limb to swell; in which case, I apprehend that gentle pressure made equally on every part of the limb, from its very extremity, would rather tend to diminish than to augment inflammation: it would form an impediment to the passage of an increased quantity of blood into the vessels of the limb, whilst it could not at all obstruct the exit of blood from them; and such being the case, it would (as it appears to me) be a means of lessening rather than of aggravating inflammation. But should, however, the pressure become painful, from a disposition on the part of the limb to swell, there could be no difficulty in removing the incrustation and bandage, and again putting up the limb with a degree of pressure on it which could be comfortably borne: it certainly would be more troublesome than the loosening of a pair of splints; but I believe, from the trial of the practice which I have made upon brutes, that it will be seldom or never necessary to meddle with a limb after the incrustation shall have been made around it."

As an additional inducement to treat fractures in the manner proposed, Mr. Beaumont urges that, by making a firm incrustation around a broken leg, be the fracture simple or compound, the patient may safely, with assistance, turn occasionally from side to side, or be moved from one bed to another. This would be an important advantage, especially when injury of the soft parts of the body, from continued pressure arising from the patient's lying constantly in the same position, is to be apprehended: and, again, by "thus treating compound fractures, we obtain the advantage of so perfectly excluding the atmosphere from the injured parts, as to render the process of union as rapid as in simple fractures."

Where the soft parts of the fractured limb are so much bruised or lacerated as to render their mortification to considerable extent almost inevitable, this method of treatment is not advised.

Mr. Beaumont relates two experiments which he made upon rabbits, for the purpose of shewing the success of the practice he recommends. We see no reason why it should not be employed in many cases of fracture, and we can easily conceive that, under certain circumstances, it may be very superior to the ordinary modes of securing fractured limbs. Every practical surgeon must have felt the difficulty of keeping a fractured limb in the most favorable position by means of splints, when he had for his patient a restless child, or an irritable or perhaps insane adult; and, in either of these cases, Mr. Beaumont's plan would probably be more successful than any other yet devised. To what extent this practice can be usefully adopted, experience must determine. As far as we can judge from Mr. B.'s description of his mode of applying the case of plaster of Paris, it seems inferior, in some respects, to the plan of the Arabian surgeons, to which we have before referred, and with which he was no doubt unacquainted.

---

